# Supplementary material for: Impact of culture on refugee women’s conceptualization and experience of postpartum depression in high-income countries of resettlement: A scoping review
Source: PLoS One. 2020 Sep 1;15(9):e0238109. doi: 10.1371/journal.pone.0238109 (PMC7462258; doi:10.1371/journal.pone.0238109)
Supplement: S1 Table — (DOCX) [file pone.0238109.s002.docx]

**Appendix A**

**Search Strategies**

| **Database** | **Search Date** | **Search** |
| --- | --- | --- |
| **PsycINFO (Ovid)** | July 14, 2019 | 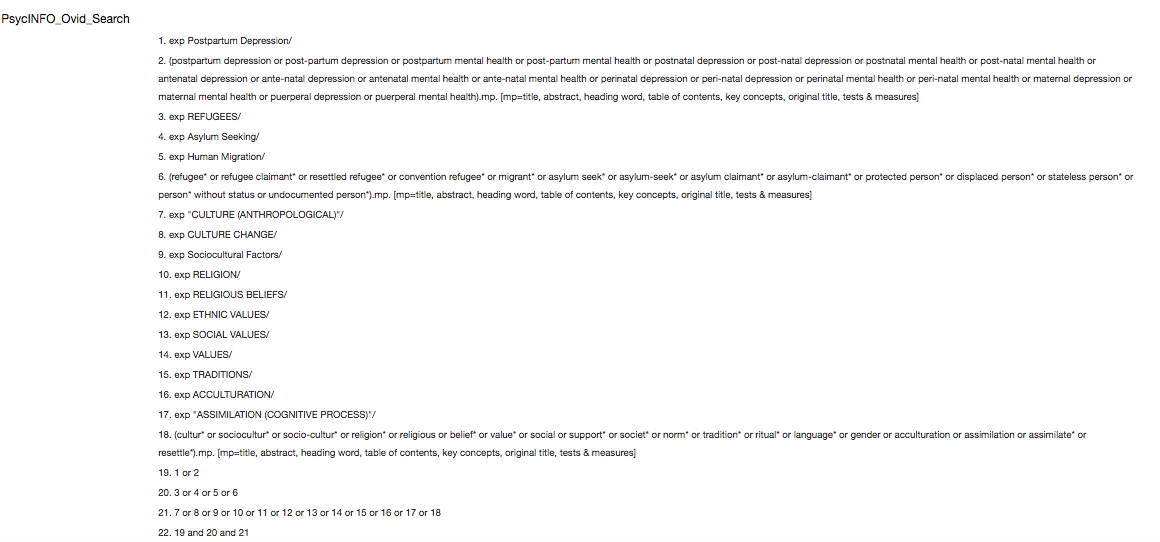 |
|  |  |  |
| **MEDLINE (Ovid)** | July 14, 2019 | 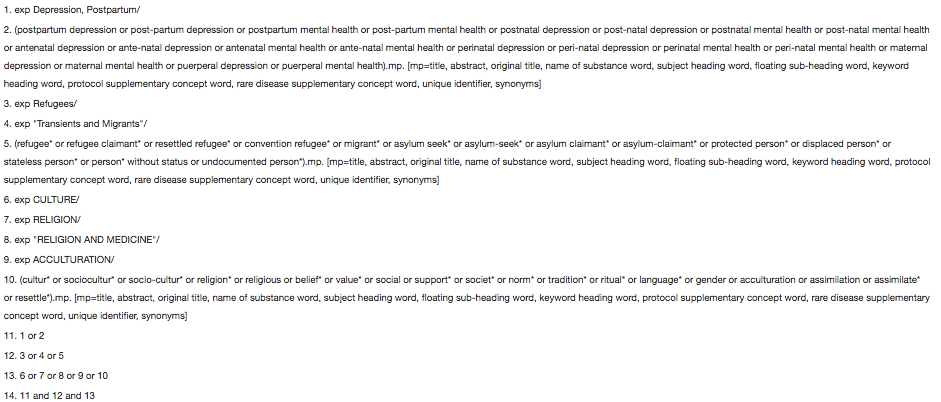 |
|  |  |  |
| **CINAHL** | July 31, 2019 | 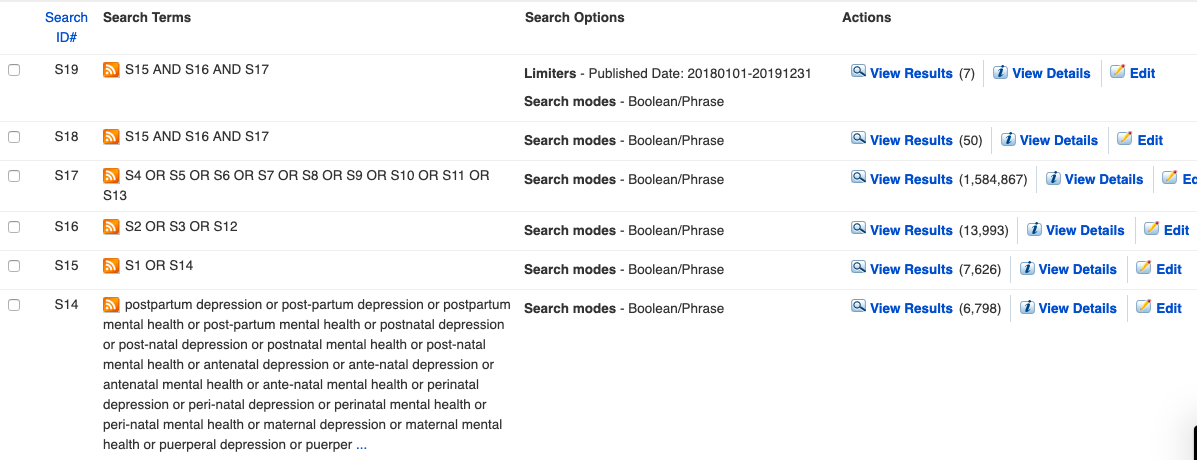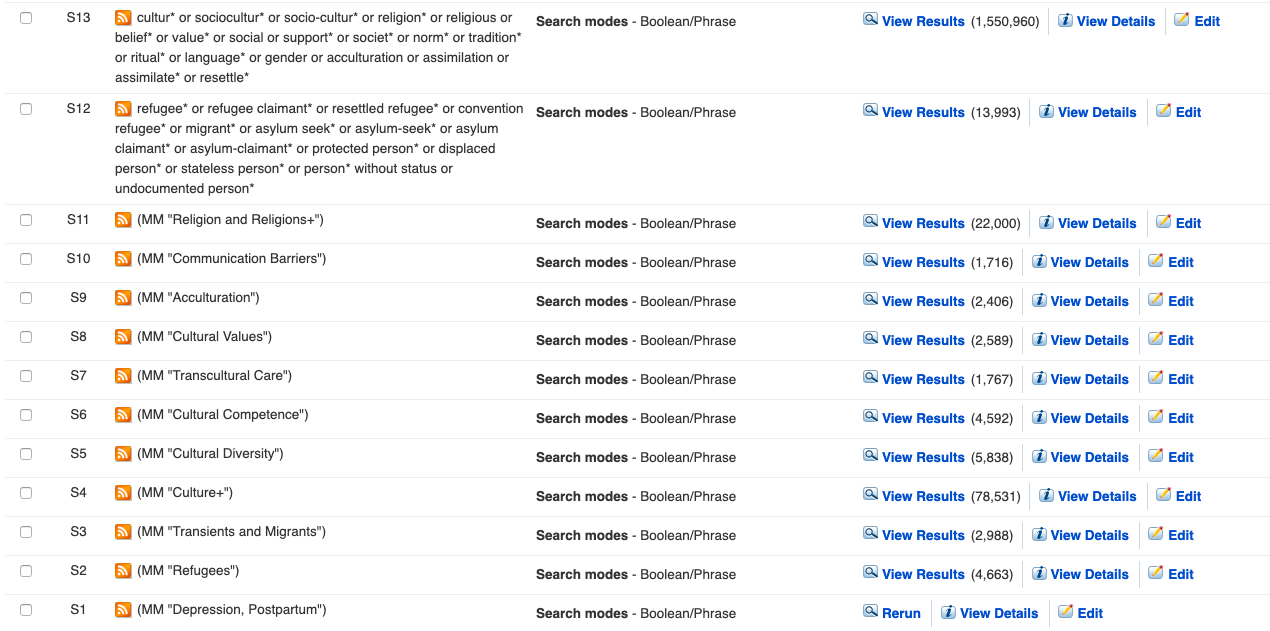 |
|  |  |  |
| **SOCINDEX** | Aug 28, 2019 | 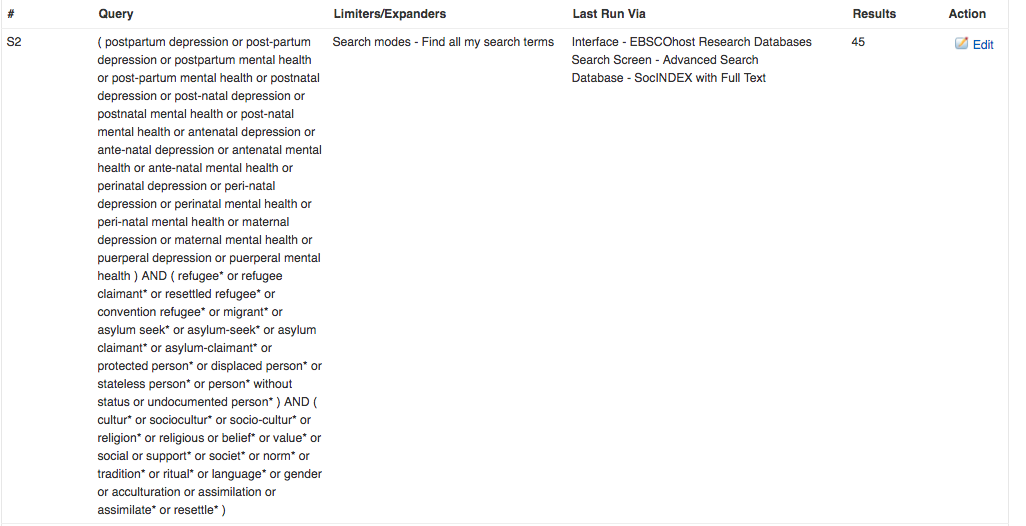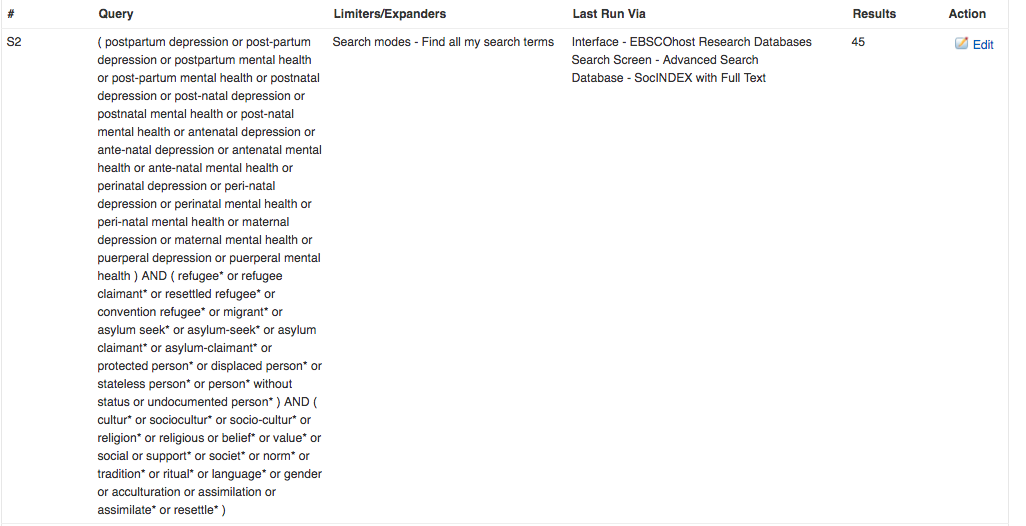 |
|  |  |  |
| **Sociological Abstracts** | July 31, 2019 | **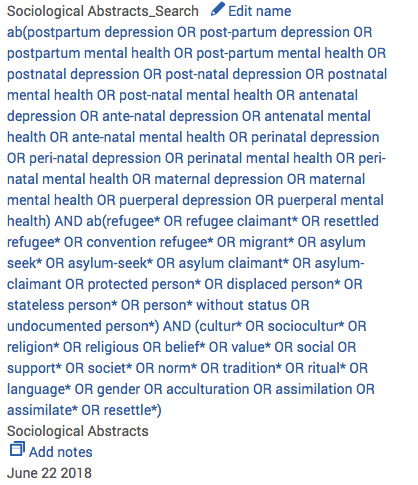** |
|  |  |  |
| **Social Sciences Citation Index** | July 31, 2019 | **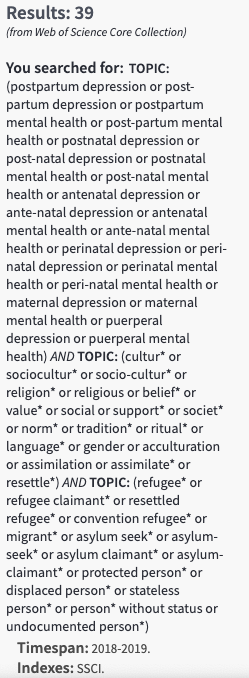** |
|  |  |  |
