# Supplementary material for: Impact of culture on refugee women’s conceptualization and experience of postpartum depression in high-income countries of resettlement: A scoping review
Source: PLoS One. 2020 Sep 1;15(9):e0238109. doi: 10.1371/journal.pone.0238109 (PMC7462258; doi:10.1371/journal.pone.0238109)
Supplement: S2 Table — (DOCX) [file pone.0238109.s003.docx]

**Appendix B**

Data collection form

| Title |  |
| --- | --- |
| Study ID (Covidence) |  |
| Journal |  |
| Publication Year |  |
| Volume/Issue |  |
| Pages |  |
| Notes: | |

# General Information

| Date form completed *(dd/mm/yyyy)* |  |
| --- | --- |
| Name of person extracting data |  |
| Notes: | |

# Study eligibility

| Study Characteristics | Eligibility criteria  *(Insert inclusion criteria for each characteristic as defined in the Protocol)* | | Eligibility criteria met? | | | Location in text or source *pg. /fig/table/other* |
| --- | --- | --- | --- | --- | --- | --- |
|  |  | | Yes | No | Unclear |  |
| Type of study | Either primary research or grey literature source | |  |  |  |  |
| Participants | Refugee, refugee claimant and/or asylum-seeking women | |  |  |  |  |
|  | Distinct analysis for refugees/asylum-seekers as sub-group | |  |  |  |  |
| Location | High-income country | |  |  |  |  |
| Focus | Experience of postpartum depression | |  |  |  |  |
|  | Analysis of cultural factors impacting postpartum depression experience | |  |  |  |  |
| INCLUDE | | EXCLUDE | | | | |
| Reason for exclusion |  | | | | | |
| Notes: | | | | | | |

**DO NOT PROCEED IF STUDY EXCLUDED FROM REVIEW**

**Full-Text Data Extraction**

## **Characteristics of Included Studies**

|  | **Descriptions as stated in report/paper** | | **Location in text or source** *pg. & fig/table/other* |
| --- | --- | --- | --- |
| Aim of the study |  | |  |
| Design *(e.g. qualitative, quantitative, prospective, cross-sectional)*  Include method of data collection (e.g. focus group discussions, interviews, questionnaires) |  | |  |
| Discipline *(e.g. Nursing, Sociology)* |  | |  |
| Duration of participation *(from recruitment to last follow-up)* |  | |  |
| Analytical strategy *(e.g. framework, theory)* |  | |  |
| Ethical approval needed/ obtained for study | Yes No Unclear |  |  |
| **Notes:** | | | |

## Participants (baseline demographics)

|  | Description  *Include comparison group data if available* | | Location in text or source *pg. & fig/table/other* |
| --- | --- | --- | --- |
| Study setting |  | |  |
| Inclusion criteria |  | |  |
| Exclusion criteria |  | |  |
| Sampling strategy *(e.g. phone, mail, clinic patients)* |  | |  |
| Informed consent obtained | Yes No Unclear |  |  |
| Total no. (n) |  | |  |
| Withdrawal (%) |  | |  |
| Exclusion (%) |  | |  |
| Attrition (%) |  | |  |
| Age (mean years) |  | |  |
| Relationship status (% with partner support) |  | |  |
| Race/Ethnicity (mean %) | White:  Black:  East Asian:  South Asian:  Middle Eastern:  Hispanic:  Other: | |  |
| Country of origin (list numerically) |  | |  |
| Time since resettlement |  | |  |
| Refugee camp exposure (yes/no) |  | |  |
| Duration of migration journey |  | |  |
| Socioeconomic status (mean $) |  | |  |
| Education level (mean last completed) |  | |  |
| Religion (mean %) | Christianity:  Islam:  Hinduism:  Buddhism:  Sikhism:  Other (specify):  Not specified: | |  |
| **Postpartum depression-specific data** | | | |
|  | Description | | Location in text or source *pg. & fig/table/other* |
| Comorbidities (mean %) |  | |  |
| PPD diagnosis data |  | |  |
| Parity before PPD diagnosis |  | |  |
| Time between resettlement and PPD diagnosis (mean) |  | |  |

## Cultural Factors Data

|  | Description as stated in report/paper | Location in text or source *pg. & fig/table/other* |
| --- | --- | --- |
| Definition of culture: |  |  |
| Key themes |  |  |
| Cultural factors identified (native culture)  - Impact (positive, neutral, negative or unknown) |  |  |
| Cultural factors identified (host culture)  - Impact (positive, neutral, negative or unknown) |  |  |
| Notes: | | |

## Other

| Study funding sources *(including role of funders)* |  |  |
| --- | --- | --- |
| Possible conflicts of interest *(for study authors)* |  |  |
| Notes: | | |

# Risk of Bias Assessment

| Domain | Risk of bias | | | Support for judgement  *(include direct quotes where available with explanatory comments)* | Location in text or source *pg. & fig/table/other* |
| --- | --- | --- | --- | --- | --- |
|  | Yes | No | Unclear |  |  |
| Inclusion & exclusion criteria specified *(yes if state specifically in text)* |  |  |  |  |  |
| Sample described *(yes if author gives information about participants AND where they were recruited from)* |  |  |  |  |  |
| Consecutive patients used *(yes if stated specifically in text)* |  |  |  |  |  |
| Referred population *(yes if stated specifically in text)* |  |  |  |  |  |
| Demographics described *(yes if more than age given)* |  |  |  |  |  |
| Explicit criteria used for postpartum depression diagnosis confirmation *(e.g. EPDS)* |  |  |  |  |  |
| Potentially important baseline differences/confounders identified |  |  |  |  |  |
| Other bias |  |  |  |  |  |
| Notes: | | | | | |

# Other Information

|  | | **Description as stated in report/paper** | **Location in text or source** *pg. & fig/table/other* | |
| --- | --- | --- | --- | --- |
| Key conclusions of study authors | |  |  | |
| References to other relevant studies | |  |  | |
| Correspondence required for further study information *(from whom, what and when)* | |  | |  |
| Recommendations for clinical practice |  | |  | |
| **Notes:** | | | | |
