# Supplementary material for: Impact of culture on refugee women’s conceptualization and experience of postpartum depression in high-income countries of resettlement: A scoping review
Source: PLoS One. 2020 Sep 1;15(9):e0238109. doi: 10.1371/journal.pone.0238109 (PMC7462258; doi:10.1371/journal.pone.0238109)
Supplement: Attachment — Submitted filename: Peer Review.pdf [file pone.0238109.s004.pdf]

Line 50
Please clarify - 'highly distinct' - I think you may mean very different?

Line 54
Does this also include undocumented migrant women?

Line 96
Add a definition of refugee in this section (eg: how does UNHCR define) and how you are including asylum seeking and refugee claimants within this definition (and undocumented migrants?) to provide context to your review.

Line 100
This is a long sentence, please clarify to highlight main ideas.

Line 116-121
This sentence is too long, please re-organize.

Line 121
The aims of scoping reviews as outlined by JBI include identifying types of evidence in the field, identifying knowledge gaps, clarifying key concepts, looking at how research has been done in the substantive area you speak of (ie: refugee women and the cultural conceptualizations and experiences of PPD), and to locate central characteristics related to the concepts of refugee maternal mental health. Integrating these aims into your objectives and into why you chose a JBI scoping review would help with methodological alignment.

Is there a reason why you selected a scoping review over a systematic review?

Line 128
Can you define *cross-culturalism* a bit more please? Where does it come from; where do you draw this concept from and how is it conceptualized within this study.

Line 148
Please include this in your introduction.

Line 155
With the history of migrant discourse being steeped in conflation of terms, I wonder if excluding studies with immigrant/migrant foci inadvertently excluded

studies with refugee women experiences of PPD... Perhaps this can be discussed and elaborated on in your limitations section.

Please specify inclusion/exclusion of refugee women and their location within the PPD care provision process. For example, were refugee women who had already been diagnosed specifically with PPD included or those who had tested high on screening tools only, or...

Line 182
Were there any publication limits? (ie: date limitations)

Line 211
How were disagreements (if there were any) resolved during study selection process?

Line 225
Please state how the grey literature you gathered informed or was integrated into your scoping review

Line 427
Long sentence with multiple findings - please explicate to clarify and highlight these findings.

Line 534
Is this because the focus of this study was on cultural conceptualizations rather than how culture and migration status intermingle to shape conceptualizations? The unique journeys of refugees and the sociopolitical and economic determinants that shape refugee conceptualizations of PPD could be further integrated to capture the differences you hypothesized about.

Line 538
Factors not being considered here include amount of time a woman has lived in their high-income host country which impacts "cross-cultural' conceptualization, integration and assimilation. What also is not being addressed within this discussion is how refugee migration journeys include living in transition. This involves living in refugee camps or communities for several years located in low or middle income countries before they are permanently allocated a country to settle in. There is literature out there that shows how pre-migration journeys among refugees affect cultural assimilation, integration and conceptualizations of mental health. This is a huge difference between a migrant who chooses to directly move

from their country of origin to a high income country. I would thus avoid extrapolating results from this scoping review to the broader migrant literature that has very different definitions and histories behind their participants. I also recommend not deducing the limited value of migration status on cultural inquiries. Although you are cautioning readers "against making the conclusion that stratification by migrant type is not of value" for a couple reasons, the idea of questioning the inextricable link of migration status to other social locations such as culture is still presented as a valid one. In doing so, unilateral understandings of human experience is being suggested along with compartmentalizing a person rather than viewing them as whole beings.

Line 546
Making final conclusions is not the aim of scoping reviews anyway, but rather recommending <u>where the gaps are in the research you have reviewed</u>.

Line 563
Great contribution to the interplay of culture and gender on post birth and resettlement transitions.

Line 590
Did these shortcomings include language barriers? Please elaborate.

Line 597
How is this ideology (individualism) defined within this review? It's a matter rooted in capitalism and necessary to elaborate on within the context of cultural diversity.

Line 600
Great thought and recommendation - are there any practical suggestions as to how healthcare providers can do so?

Line 616
I question generalizability as a goal of scoping reviews - take a look at what JBI says re: goals/aims/objectives of scoping reviews

Line 630
To be consistent with your initial inclusion criteria, you may want to change "peripartum and postpartum period" to "of childbearing age" or vice versa... I'm now thinking the use of "childbearing age" denotes prenatal care as well - please clarify.
