# Supplementary material for: Impact of culture on refugee women’s conceptualization and experience of postpartum depression in high-income countries of resettlement: A scoping review
Source: PLoS One. 2020 Sep 1;15(9):e0238109. doi: 10.1371/journal.pone.0238109 (PMC7462258; doi:10.1371/journal.pone.0238109)
Supplement: Attachment — Submitted filename: Haque - Response to Reviewers.docx [file pone.0238109.s005.docx]

**Response to Reviewers**

**Reviewer #1**:

The authors undertook a scoping review to assess the impact of culture on the conceptualization and experience of postpartum depression (PPD) by refugee or asylum-seeking women resettled in developed countries. The PRISMA guidelines for scoping reviews has been followed. The search terms "postpartum depression" in Table 2 is repeated.

**Response:** The second postpartum depression was meant to be hyphenated – this has been corrected.

**Why was the review limited to only developed countries? Can the authors provide more justification for the choice?**

**Response:** Many thanks for this comment. We have elaborated on our justification for why we chose to limit this review to only high-income countries in the second to last paragraph of our introduction which now reads as follows: *A focus on refugee women resettled in high-income countries has been chosen for 2 main reasons. Firstly, refugee women resettled in high-income countries face a high risk for developing PPD which drives our interest in better understanding this phenomenon. Secondly, increasing numbers of refugee women of childbearing age are resettling in high-income countries. As such, it is imperative we gain a better understanding of how culture impacts the ways in which refugee women understand and describe their postpartum experiences when resettling in high-income countries, as well as how our own culture (and interface between our culture and that of our patients) impacts how we assess, diagnose and treat PPD in refugee women.*

**Reviewer #2**:

Hypothesis: There are important differences in the conceptualization and experiences of PPD between refugee women and other migrant women which have yet to be clearly defined.

Objective: to characterize what is currently known in the literature about the impact of culture on the conceptualization and experience of PPD in refugee women resettled in high-income countries.

The authors undertook a scoping review justifying that it is the preferred methodological framework due to the expected heterogeneity in the current research disciplines and methodologies addressing this question.

Overall, the manuscript is well written. However, it could benefit with concision, including reporting some of the key findings diagrammatically or in table, if possible.

**Response:** Many thanks for the suggestion. In response, we have added a table summarizing our key findings divided along the lines of the four themes that emerged from the review.

**Reviewer 1&2 Comments**

**Abstract**

Line 48: may be omit “will”

**Response:** “Will” has been omitted.

Briefly report the eligibility criteria for including studies.

**Response:** Further elaboration on the eligibility criteria for including studies has been added: *Studies were eligible if they (1) focused on refugee women who had a pregnancy during forced migration or upon resettlement in a high-income country and (2) focused on the impact of culture on women’s conceptualization and/or experience of PPD.*

**Introduction**

Introduction is written well and provides strong rationale for the scoping review.

Line 115-116: It is not clear what the following statement means. “However, a focus on the sub population of refugee women is often lacking.

**Response**: With this sentence, we are explaining that studies tend to analyze migrants as one unified group rather than doing sub-analyses or paying special attention to sub-populations of migrants (such as, for example, economic migrants, refugees, or migrants resettled for family reunification). We have adjusted the sentence so that this point made more clear: *However, studies tend to group all subtypes of migrants together and consequently lack special attention to the sub-population of refugee women.*

Line 127: Need reference.

**Response:** This sentence was adjusted, and a reference was added: *Secondly, increasing numbers of refugee women of childbearing age are resettling in high-income countries (17)*

Line 128-131: Please consider revising. I would say, we assessed impact of culture on the conceptualization and experience of PPD in refugee women, and what are the implications for diagnosing and managing PPD…

**Response:** The lines were adjusted as per your suggestion: *As such, it is imperative we gain a better understanding of how culture impacts the ways in which refugee women understand and describe their postpartum experiences when resettling in high-income countries, as well as how our own culture (and interface between our culture and that of our patients) impacts how we assess, diagnose and treat PPD in refugee women.*

**Methods**

Line 140-143: I think these lines are redundant. Objectives are rationale are covered in the Introduction.

**Response:** Thank-you for this comment. We agree. These lines were removed.

Line 167-168: Please consider revising. “was their” is not going well.

**Response:** This sentence was revised to the following: *The outcome measure for our research question was the refugee woman’s conceptualization and/or experience of PPD.*

**Results**

Line 293-295: should this be part of theme 3?

**Response:** Yes, we think you have a raised a good point and these lines better fit with theme 3. As such, it has been moved there: *In Ahmed et al.’s study, strong support provided around the time of birth was protective against depressive feelings and a lack of family support was a stressor in the postpartum period for several Syrian refugee women (2017).*

Line 318-383: provide reference(s)

**Response:** References in the results section were added for each sentence that directly discussed the included article.

Line 385-386: provide reference(s)

**Response:** References were added.

**Limitations**

It seems, refugees in LMICs were excluded, assuming that the culture may not be different from the host country. This may be not entirely true. Consider mentioning this in Limitations.

**Response:** Thank-you for this comment. We have decided that this is an important point which requires mention and clarification in the Introduction. As such we have clarified our reasoning for including high income countries (and therefore excluding LMICs) in the Introduction and have also made mention of this in the Limitations section. Sentences that have been added to the introduction read as follows: *A focus on refugee women resettled in high-income countries has been chosen for 2 main reasons. Firstly, refugee women resettled in high-income countries face a high risk for developing PPD which drives our interest in better understanding this phenomenon. Secondly, increasing numbers of refugee women of childbearing age are resettling in high-income countries.* *As such, it is imperative we gain a better understanding of how culture impacts the ways in which refugee women understand and describe their postpartum experiences when resettling in high-income countries, as well as how our own culture (and interface between our culture and that of our patients) impacts how we assess, diagnose and treat PPD in refugee women.*

Sentences added to the limitations read as follows: *Further research is needed to better understand the broad array of conceptualizations and experiences of PPD held by refugee women from and resettling in diverse countries and cultures around the world which are not yet represented in the literature. This includes a closer examination of the impact of culture on the conceptualizations and experiences of PPD of refugee women resettled in low- and middle-income countries which were excluded from this review.*

**Reviewer #3:** This scoping review is a needed contribution within refugee maternal mental health scholarship. Please take a look at what JBI says regarding the intentions of a scoping review to align with your goals/objectives. I've made some other recommendations and wish you all the best in your work with refugee women. Thank you for bringing to light the need for more inquiry within this often-overlooked population.

**Response**: Many thanks for your kind words in support of our manuscript. We greatly appreciate your feedback and recommendations.

**Reviewer 3 Comments**

Line 50

Please clarify - 'highly distinct' - I think you may mean very different?

**Response:** Yes, we did mean very different and agree this would increase the clarity of the sentence. As such, the sentence has been corrected to: *Approximately 16% of global refugees resettle in high-income countries which are often culturally very different from their home countries.*

Line 54

Does this also include undocumented migrant women?

**Response:** The definition of refugee can include undocumented migrants. We included refugee claimants and asylum seekers, that fit in this definition of undocumented migrants however, we did not include individuals who were not seeking refugee status, those who were not granted permission to stay within a country, and those who overstayed their visa. To clarify this concept, we have added a definition of undocumented migrants and specified what subgroup of undocumented migrants we included in the Eligibility Criteria: *Refugee claimants and asylum seekers are classified as undocumented migrants (22). Undocumented migrants also include those who overstay their visas, and those who are not seeking refugee status but stay within a country without permission – these individuals were not included in the scoping review (22).*

Line 96

Add a definition of refugee in this section (eg: how does UNHCR define) and how you are including asylum seeking and refugee claimants within this definition (and undocumented migrants?) to provide context to your review.

**Response:** Thank-you for the suggestion. A UNHCR definition of “refugee” was added to the manuscript: *The UNHCR define refugees as people “who have fled war, violence, conflict or persecution and have crossed an international border to find safety in another country”, while asylum seekers (also known as refugee claimants) are people who have made in-country claims for asylum that have yet to be processed (2,3).*

Line 100

This is a long sentence, please clarify to highlight main ideas.

**Response:** The sentence was broken up into smaller sentences and now reads as follows: *One such adverse outcome is postpartum depression (PPD). Studies demonstrate a 2-5 times greater risk of refugee women resettled in high-income countries developing PPD compared to non-migrant women (10,11).*

Line 116-121

This sentence is too long, please re-organize.

**Response:** Thank-you for the feedback. The sentence was broken up into smaller sentences: *Refugee women, however, may face unique challenges compared to women with other migration journeys due to the forced nature of their migration, as well as other factors that have yet to be fully understood. An examination of available evidence regarding the impact of culture on the conceptualization and experience of PPD which focuses on refugee women resettled in high-income countries is needed.*

Line 121

The aims of scoping reviews as outlined by JBI include identifying types of evidence in the field, identifying knowledge gaps, clarifying key concepts, looking at how research has been done in the substantive area you speak of (ie: refugee women and the cultural conceptualizations and experiences of PPD), and to locate central characteristics related to the concepts of refugee maternal mental health. Integrating these aims into your objectives and into why you chose a JBI scoping review would help with methodological alignment.

**Response:** We appreciate the feedback. As such, we have revised the last 2 paragraphs of our introduction to more clearly frame our research objectives, which provides a clearer justification for our decision to conduct a JBI scoping review. This paragraph now reads as follows: *As such, the objective of our study is to provide a broad and inclusive overview of available evidence regarding the impact of culture on the conceptualization and experience of PPD amongst refugee women resettled in high-income countries. Such an overview will enable us to clarify key concepts and findings in the literature, while also identifying important knowledge gaps to guide future research aimed at improving clinical detection and management of PPD amongst refugee women resettled in high-income countries. In order to achieve these research objectives a scoping review was conducted in accordance with JBI’s Methodology for Scoping Reviews.*

Is there a reason why you selected a scoping review over a systematic review?

**Response:** As mentioned above, we have revised the last 2 paragraphs of our introduction to more clearly frame our research objectives, which provides clearer justification for our decision to conduct a scoping review over a systematic review. The most relevant paragraph now reads as follows: *As such, the objective of our study is to provide a broad and inclusive overview of available evidence regarding the impact of culture on the conceptualization and experience of PPD amongst refugee women resettled in high-income countries. Such an overview will enable us to clarify key concepts and findings in the literature, while also identifying important knowledge gaps to guide future research aimed at improving clinical detection and management of PPD amongst refugee women resettled in high-income countries. In order to achieve these research objectives a scoping review was conducted in accordance with JBI’s Methodology for Scoping Reviews.*

Line 128

Can you define cross-culturalism a bit more please? Where does it come from; where do you draw this concept from and how is it conceptualized within this study.

**Response:** We have defined cross culturalism within this study as it is described in the discipline of cross-cultural psychology and have added a sentence which highlights this definition in our manuscript: *Here, we define cross-culturalism drawing from the definition used in the discipline of cross-cultural psychology whereby cross-culturalism is the “similarities and differences in behavior among individuals who have developed in different cultures.”*

Line 148

Please include this in your introduction.

**Response**: We included the following sentence in the introduction: *Our study population of interest was women identified as refugees, refugee claimants or asylum seekers who had a pregnancy during forced migration or upon resettlement.*

Line 155

With the history of migrant discourse being steeped in conflation of terms, I wonder if excluding studies with immigrant/migrant foci inadvertently excluded studies with refugee women experiences of PPD... Perhaps this can be discussed and elaborated on in your limitations section.

**Response**: Thank-you for your comment. We realize this is a limitation of our study and have worked to elaborate on this in our limitations section by stating the following: *Due to our criteria that articles include an explicit sub-analysis of refugee women’s cultural conceptualization and/or experience of PPD, studies that included refugee women under the broader categorization of “migrant” or “immigrant” women were excluded. We chose this approach as such studies did not provide findings stratified by migrant type, making it impossible to extract data from these studies that were specific to refugee women. Further, our search strategy specified that articles use the term “refugee” (and analogous terms as outlined in our search strategy* *in Table 2). This may have inadvertently excluded studies of women who fit the definition of “refugee” if this specific term or analogous terms were not explicitly used. This question arose, for example, when screening studies of Hispanic and Latino women resettled in the United States, in which it was not always clear (and often not explicitly stated) whether they were refugee women or not.*

Please specify inclusion/exclusion of refugee women and their location within the PPD care provision process. For example, were refugee women who had already been diagnosed specifically with PPD included or those who had tested high on screening tools only, or...

**Response**: We included the following clarifications on this topic in the Participants section: *The definition of PPD diagnosis was inclusive to women who had a previous diagnosis of PPD confirmed by screening tools or clinical depression. We also included studies where women with previous pregnancies discussed their understanding of PPD if the study included women with PPD symptomatology.*

Line 182

Were there any publication limits? (ie: date limitations)

**Response**: There were no specific publication limits applied to our search criteria. To ensure clarify, we have added the following sentence to the section entitled “Eligibility Criteria”: *No restriction was placed on study methodology, language of publication or date of publication.*

Line 211

How were disagreements (if there were any) resolved during study selection process?

**Response**: Disagreements were discussed by both reviewers and included studies were finalized together – the following has been added to Study Selection. *Disagreements were resolved through discussion between both reviewers (SH, MM).*

Line 225

Please state how the grey literature you gathered informed or was integrated into your scoping review.

**Response**: Thank-you for this comment as we did not make this clear in our initial manuscript. The grey literature did not meet inclusion criteria. We have included this in the first paragraph of the results section to make this clear: *No studies identified through the search of grey literature met inclusion criteria.*

Line 427

Long sentence with multiple findings - please explicate to clarify and highlight these findings.

**Response**: The sentence has now been broken into smaller sentences and further clarity has been provided. The sentences highlighting these findings now reads as follows: *Nakash et al. (2016) provide another angle on this topic in their study examining the association between PPD, acculturation and the mother-infant bond among Eritrean asylum seekers in Israel. As part of this study they asked women to rate the status of their current romantic relationship (using a 5-point Likert scale ranging from 1 “unhappy/unstable” to 5 “loving and stable”) and also had women complete the Maternal Postnatal Attachment Scale (MPAS), a self-reported measure of mother-to-infant boding during the infant’s first year of life. What they found was that higher quality of current romantic relationship predicted a higher self-reported mother-to-infant bond. This finding supports the notion that having a romantic partner that is perceived as loving and supportive is beneficial for refugee mothers and babies in their countries of resettlement and highlights the important role partners play in the peripartum period of refugee women at a time when social supports, including female kinship, are often lacking.*

Line 534

Is this because the focus of this study was on cultural conceptualizations rather than how culture and migration status intermingle to shape conceptualizations? The unique journeys of refugees and the sociopolitical and economic determinants that shape refugee conceptualizations of PPD could be further integrated to capture the differences you hypothesized about.

Line 538

Factors not being considered here include amount of time a woman has lived in their high-income host country which impacts "cross-cultural' conceptualization, integration and assimilation. What also is not being addressed within this discussion is how refugee migration journeys include living in transition. This involves living in refugee camps or communities for several years located in low- or middle-income countries before they are permanently allocated a country to settle in. There is literature out there that shows how pre-migration journeys among refugees affect cultural assimilation, integration and conceptualizations of mental health. This is a huge difference between a migrant who chooses to directly move from their country of origin to a high income country. I would thus avoid extrapolating results from this scoping review to the broader migrant literature that has very different definitions and histories behind their participants. I also recommend not deducing the limited value of migration status on cultural inquiries. Although you are cautioning readers "against making the conclusion that stratification by migrant type is not of value" for a couple reasons, the idea of questioning the inextricable link of migration status to other social locations such as culture is still presented as a valid one. In doing so, unilateral understandings of human experience is being suggested along with compartmentalizing a person rather than viewing them as whole beings.

**Response to Line 534 & 538 comments:** Reviewers have raised some important points about other key factors that impact refugee women’s conceptualizations and experiences of PPD beyond culture and migration type. We have reflected on this and revised the paragraph in our discussion section which highlights this point and also mention it in our limitations section. The paragraph in our discussion section is written now as follows: *The thematic similarities found across the literature of PPD in migrant women post-resettlement raises the question of whether or not it is imperative that we stratify migrant women by migration type in studies on PPD, as has been suggested (and as we suspected) (30). Perhaps when it comes to looking specifically at the impact culture has on the conceptualization and experience of PPD, it is a woman’s culture and its contrast with that of the culture of the country of resettlement that matters and not her migration status. This is plausible. However, we would caution against making the assumption that stratification by migrant type is not of value for three key reasons. Firstly, our review identified only 8 studies representing approximately 300 refugee and asylum-seeking women resettled in high-income countries around the world. To make any final conclusion on PPD in refugee populations with such a limited evidence base would be premature. Secondly, a number of studies of PPD amongst migrant women included refugee women but made no distinction between these migrant populations making it difficult to know which migrant sub-type was driving the findings. Stratification would have shed light on this. Thirdly, there are likely other key factors beyond culture that impact refugee women’s conceptualization and experience of PPD, which have not been explored through this scoping review. Such factors include duration of migration journey, time spent in a neighboring low- or middle-income country or refugee camp before resettling in their host country, and time since arrival in host country. The sociopolitical and economic climates of a woman’s country of origin and host country may also play an important role. Research that distinguishes between migrant type (i.e. economic or family migrant vs. refugee vs. asylum-seeker) and better characterizes the migration journey is needed before any conclusion can be made about how to best stratify data on migrant women in research surrounding the topic of PPD.*

Line 546

Making final conclusions is not the aim of scoping reviews anyway, but rather recommending where the gaps are in the research you have reviewed.

**Response:** Thank-you for this comment. We changed the wording throughout the discussion to more clearly represent that we are not aiming to draw any final conclusions but instead highlight what we know based on the current literature and what future research is needed. Here is an example of one change: *Further research is needed to better understand the broad array of conceptualizations and experiences of PPD held by refugee women from diverse countries and cultures around the world not yet represented in the literature.*

Line 563

Great contribution to the interplay of culture and gender on post birth and resettlement transitions.

**Response:** Thank you!

Line 590

Did these shortcomings include language barriers? Please elaborate.

**Response:** Yes, these shortcoming did include language barriers. To address this we have added examples to the sentence as follows: *Health care providers should reflect on their current use of this tool to ensure it is being used with an awareness of its shortcomings (e.g. cultural and linguistic misunderstandings in regard to certain EPDS items).*

Line 597

How is this ideology (individualism) defined within this review? It’s a matter rooted in capitalism and necessary to elaborate on within the context of cultural diversity.

**Response**: We have added definitions of individualism and collectivism in the Discussion section to help clarify what we mean by these terms: *Individualism is defined as “a social pattern that consists loosely linked individuals who view themselves as independent of collectives; are primarily motivated by their own preferences, needs, rights … [they] give priority to their personal goals over the goals of others; and emphasize rational analyses of the advantages and disadvantages to associating with others.” This cultural framework may stand in stark contrast to the views of the collectivist cultures of many of our refugee patients. Unlike individualism, collective is “a social pattern consisting of closely linked individuals who see themselves as parts of one or more collectives … [they] are primarily motivated by the norms of, and duties imposed by, those collectives …”.*

Line 600

Great thought and recommendation - are there any practical suggestions as to how healthcare providers can do so?

**Response:** We added the practical recommendation of cultural competency training: *Engaging in cultural competency training during medical school or through continuing professional development for health care providers already in practice in one way that this critical refection and exploration could be achieved.*

Line 616

I question generalizability as a goal of scoping reviews - take a look at what JBI says re: goals/aims/objectives of scoping reviews.

**Response**: Many thanks for your comment about generalizability as a goal of scoping reviews. We have clarified our study objectives as outlined in the point above and have also taken out comment about the limited generalizability of our study findings in the Limitations sections of our paper. Given that this is not a goal of a scoping review, it does not seem like a limitation that requires comment. It has therefore been removed.

Line 630

To be consistent with your initial inclusion criteria, you may want to change "peripartum and postpartum period" to "of childbearing age" or vice versa... I'm now thinking the use of "childbearing age" denotes prenatal care as well – please clarify.

**Response**: Thank-you for this comment. Since our initial inclusion criteria was refugee women who had a pregnancy during forced migration or upon resettlement in a high-income country we are not sure that “childbearing age” is a fitting term for our conclusion. Instead we have revised the sentence as follows to highlight the focus on pregnant and postpartum refugee women in our review: *Health care providers caring for pregnant and postpartum refugee women should acknowledge and explore the cultural implications of PPD in order to appropriately assess, diagnose and treat PPD in refugee women from diverse cultural backgrounds*.
